# Selective inhibition of spleen tyrosine kinase (SYK) with a novel orally bioavailable small molecule inhibitor, RO9021, impinges on various innate and adaptive immune responses: implications for SYK inhibitors in autoimmune disease therapy

**DOI:** 10.1186/ar4329

**Published:** 2013-10-04

**Authors:** Cheng Liao, Jonathan Hsu, Yong Kim, Dong-Qing Hu, Daigen Xu, Jun Zhang, Achal Pashine, John Menke, Toni Whittard, Natasha Romero, Theresa Truitt, Michelle Slade, Christine Lukacs, Johannes Hermann, Mingyan Zhou, Matthew Lucas, Satwant Narula, Julie DeMartino, Seng-Lai Tan

**Affiliations:** 1 Inflammation Discovery and Therapeutic Area, Hoffmann-La Roche, 340 Kingsland Street, Nutley, NJ 07110, USA; 2 Discovery Technology, Hoffmann-La Roche, 340 Kingsland Street, Nutley, NJ 07110, USA; 3 Discovery Chemistry, Hoffmann-La Roche, 340 Kingsland Street, Nutley, NJ 07110, USA; 4 Drug Metabolism and Pharmacokinetics, Hoffmann-La Roche, 340 Kingsland Street, Nutley, NJ 07110, USA; 5 Present address: EMD Serono Research & Development Institute, 45A Middlesex Turnpike, Billerica, MA 01821, USA; 6 Present address: Lilly Research Laboratories, Tailored Therapeutics Autoimmunity/Muscular and Skeletal, Indianapolis, IN 46285, USA; 7 Present address: Cubist Pharmaceuticals, 65 Hayden Avenue, Lexington, MA 02421, USA

## Abstract

**Introduction:**

Spleen tyrosine kinase (SYK) is a key integrator of intracellular signals triggered by activated immunoreceptors, including Bcell receptors (BCR) and Fc receptors, which are important for the development and function of lymphoid cells. Given the clinical efficacy of Bcell depletion in the treatment of rheumatoid arthritis and multiple sclerosis, pharmacological modulation of Bcells using orally active small molecules that selectively target SYK presents an attractive alternative therapeutic strategy.

**Methods:**

A SYK inhibitor was developed and assayed in various *in vitro* systems and in the mouse model of collagen-induced arthritis (mCIA).

**Results:**

A novel ATP-competitive inhibitor of SYK, 6-[(1R,2S)-2-Amino-cyclohexylamino]-4-(5,6-dimethyl-pyridin-2-ylamino)-pyridazine-3-carboxylic acid amide, designated RO9021, with an adequate kinase selectivity profile and oral bioavailability, was developed. In addition to suppression of BCR signaling in human peripheral blood mononuclear cells (PBMC) and whole blood, FcγR signaling in human monocytes, and FcϵR signaling in human mast cells, RO9021 blocked osteoclastogenesis from mouse bone marrow macrophages *in vitro*. Interestingly, Toll-like Receptor (TLR) 9 signaling in human Bcells was inhibited by RO9021, resulting in decreased levels of plasmablasts, immunoglobulin (Ig) M and IgG upon B-cell differentiation. RO9021 also potently inhibited type I interferon production by human plasmacytoid dendritic cells (pDC) upon TLR9 activation. This effect is specific to TLR9 as RO9021 did not inhibit TLR4- or JAK-STAT-mediated signaling. Finally, oral administration of RO9021 inhibited arthritis progression in the mCIA model, with observable pharmacokinetics (PK)-pharmacodynamic (PD) correlation.

**Conclusions:**

Inhibition of SYK kinase activity impinges on various innate and adaptive immune responses. RO9021 could serve as a starting point for the development of selective SYK inhibitors for the treatment of inflammation-related and autoimmune-related disorders.

## Introduction

Effective treatments of human autoimmune diseases, which are complex and heterogeneous by nature, require therapeutic perturbation or restoration of multiple redundant and distinct mechanisms, or a master regulator of such pathways. In the case of rheumatoid arthritis (RA) pathogenesis, the critical role of the adaptive immune response and proinflammatory cytokines has been unequivocally established by the efficacy of marketed biologics targeting tumor necrosis factor (TNF) alpha, interleukin (IL)-6, CD20 (B-cell depletion) and CD80/86 (modulation of T-cell co-stimulation). However, their efficacy are capped by limited efficacy, with 40% of patients never responding to treatments and only 20% of patients experiencing a major reduction in disease activity [1]. There thus remains a tremendous unmet clinical need for more effective therapeutic strategies, with a goal of sustained remission for a greater number of patients with RA.

Current therapeutic strategies pursued by the biopharmaceutical industry include those that target the janus kinase (JAK)-mediated signaling pathway, lymphocyte migration using chemokine CCR1 antagonist, and B cells using either depleting antibodies that recognize common cell surface antigens, such as CD22 and CD19, or blocking antibodies to B-cell survival factor such as B-cell activating factor (BAFF) or a proliferation-inducing ligand (APRIL). Although the pan-JAK inhibitor tofacitinib was recently approved by the US Food and Drug Administration for the treatment of RA, it is still not superior to the biologics in terms of efficacy and safety. For other autoimmune diseases that are in dire need of safer and/or more effective therapies, the anti-BAFF antibody belimumab, despite showing marginal efficacy in clinical trials, was approved for treatment of systemic lupus erythematous (SLE). Disappointingly, another anti-BAFF antibody (tabalumab) also did not show adequate efficacy in a phase 3 RA trial (Elli Lilly news release, 13 December 2012). Whether an agent that neutralizes both BAFF and APRIL will produce better results remains to be seen.

Other emerging approaches target key enzymes involved in mediating multiple signal transduction pathways. One such enzyme is the spleen tyrosine kinase (SYK), which is a master regulator in coupling activated immunoreceptors to the mobilization of downstream signal transduction cascades that affect diverse biological functions. One of the best characterized modules in the transmission of B-cell receptor (BCR) activating signals within B cells is the SYK–Bruton’s tyrosine kinase (BTK) axis, where BTK acts as an essential downstream effector of SYK in regulating both the maturation and survival of the B-cell lineage. Given the central role of SYK in transmission of antigen receptor signals that are critical for autoantibody production and the various innate immune effector functions, pharmacological inhibition of the catalytic function of SYK is expected to have pleiotropic anti-inflammatory effects and to impact multiple steps in the pathogenesis of autoimmune disorders [2]. This could result in greater and/or broader therapeutic efficacy as well as increased coverage of the patient population, and perhaps a decreased propensity to lose therapeutic efficacy over time.

Here, we describe the discovery and characterization of a novel ATP-competitive inhibitor of SYK: 6-[(1R,2S)-2-amino-cyclohexylamino]-4-(5,6-dimethyl-pyridin-2-ylamino)-pyridazine-3-carboxylic acid amide, designated RO9021. The inhibitor RO9021 has reasonable kinase selectivity profile, potency and oral bioavailability and is capable of suppressing various innate and adaptive immune responses *in vitro*, as well as disease progression in the mouse collagen-induced arthritis (mCIA) model. RO9021 could thus serve as a lead candidate for further development of selective SYK inhibitors for the potential treatment of immunological disorders.

## Materials and methods

### Experimental animals

C57BL/6 and DBA/1J adult mice were purchased from Charles River Laboratories (Wilmington, MA, USA). All animal procedures were approved by and conducted in accordance with the Institutional Animal Care and Use Committee guideline at Hoffmann-La Roche (Nutley, NJ, USA).

### Chemical compounds and reagents

SYK inhibitor RO9021 was designed and synthesized at Hoffmann-La Roche. Tofacitinib citrate (CP-690,550) was acquired from Selleck Chemicals LLC (Houston, TX, USA). All chemical reagents were purchased from Sigma-Aldrich (St Louis, MO, USA), antibodies for flow cytometric analysis were acquired from BD Biosciences (San Jose, CA, USA), cytokines were acquired from R&D Systems (Minneapolis, MN, USA) and antibodies for western blots were acquired from Cell Signaling Technologies (Danvers, MA, USA), unless indicated otherwise.

### SYK inhibitor, kinase selectivity and kinase activity assays

RO9021 was designed and synthesized at Hoffmann-La Roche, Inc. Specificity to SYK was assessed by an ATP competitive binding assay at 1 μM compound concentration at KINOMEscan Inc. (San Diego, CA, USA) [3].

The inhibitory potency to SYK was determined in a radiometric assay using inactive SYK kinase. Briefly, SYK protein (Invitrogen, Carlsbad, CA, USA) was dephosphorylated by PTP1B phosphatase (Invitrogen) and then the reaction was initiated by the addition of substrate cocktail that contained 20 μM ATP, 0.025 μCi ATP-γ-^33^P (Perkin Elmer, Waltham, MA, USA) and 10 μM biotinylated synthetic peptide (Biotin-EPEGDYEEVLE; Biomer Technology, Pleasanton, CA, USA) [4-6]. The reaction was carried out for 30 minutes and resulting ^33^P incorporation was determined by top counter.

### Co-crystallization of SYK and RO9021

SYK (356 to 635) containing a kinase domain was cloned, expressed, and purified, and co-crystallization of SYK and RO9021 was carried out following the protocol as reported previously by our group [7,8]. The structure has been deposited in the Protein Data Bank [PDB:4GFG].

### Calcium influx fluorometric imaging plate reading assay

Human B-cell lymphoma cell line Ramos (CRL-1596; American Type Culture Collection, Manassas, VA, USA) or T-cell lymphoma cell line Jurkat (CRL-2063; American Type Culture Collection) were loaded with calcium dye (BD Biosciences) for the assay. Baseline fluorescence was recorded for about 20 seconds followed by stimulation with mouse anti-human IgM (10 μg/ml, clone M2E6; Antibody Solutions Inc., Mountain View, CA, USA) for Ramos cells or mouse anti-human CD3 (10 μg/ml; BD Biosciences) for Jurkat cells, and the maximal fluorescent counts in each well were recorded.

### Detection of the phosphorylation of BTK, PLCγ2, ERK and AKT

Ramos cells were pretreated with RO9021 followed by stimulation with goat F(ab′)_2_ anti-human IgM (10 μg/ml; Southern Biotech, Birmingham, AL, USA). The protein phosphorylation was detected with rabbit antibodies of anti-phospho-BTK(Y223) (Epitomics Inc., Burlingame, CA, USA), anti-phospho-PLCγ2(Y1217), anti-phospho-ERK(T202/Y204) or anti-phospho-AKT(S473).

### Flow cytometric analysis of CD69 upregulation in B cells

Heparinized blood was collected from healthy volunteers and pre-incubated with RO9021 followed by stimulation with goat F(ab′)_2_ anti-human IgM (50 μg/ml) overnight. The samples were stained with PE mouse anti-human CD20 and APC mouse anti-human CD69. The percentage of activated (CD69^hi^) B cells was determined using unstimulated (negative control) and stimulated (positive control) samples as references.

### Fc receptor-mediated and lipopolysaccharide-mediated TNFα production in human monocytes

Peripheral blood mononuclear cells (PBMCs) were isolated by centrifugation from heparinized blood in a Vacutainer CPT Cell Preparation Tube (BD Biosciences). PBMCs were cultured for 1 to 2 hours to allow monocytes to adhere, and nonadherent cells were washed away. The monocytes were stimulated with human IgG-coated (Jackson Immunology, West Grove, PA, USA) copolymer microsphere beads (Thermo Scientific, Fremont, CA, USA) or lipopolysaccharide (1 ng/ml; Sigma-Aldrich) for 4 hours. TNFα levels in supernatants were determined by enzyme-linked immunosorbent assay kits (BD Biosciences).

### IgE-induced histamine release in human mast cells

The method has been reported previously by our group [9]. Briefly, human cord blood-derived CD34^+^ hematopoietic stem cells (AllCells, Emeryville, CA, USA) were cultured in a serum-free StemPro-34 medium (Invitrogen) with stem cell factor (100 ng/ml) and IL-6 (50 ng/ml) for 8 weeks followed by 5-day stimulation with IL-4 (10 ng/ml). For measuring histamine release, cells were sensitized with 0.1 μg/ml anti-4-hydroxy-3-nitrophenylacetyl hapten IgE (Serotec, Raleigh, NC, USA) overnight, and then cross-linked with 1 μg/ml NP(30)-BSA (Biosearch Technologies, Novato, CA, USA) for 30 minutes. Supernatants were collected and assayed for histamine release using a histamine enzyme immunoassay (Oxford Biomedical Research, Rochester Hills, MI, USA). The percentage of histamine release was calculated by comparing various treatments with positive control.

### Flow cytometric analysis of phosphorylated STAT1 and STAT5

Human PBMCs were pre-incubated with compound for 30 minutes followed by 20 minutes stimulation with IL-2 (100 ng/ml) for signal transducers and activators of transcription 5 (STAT5) phosphorylation or IFNγ (100 ng/ml) for STAT1 phosphorylation. For IL-2-induced STAT5 phosphorylation, cells were stained with FITC anti-human CD3 and Alexa Fluor 647 anti-STAT5(pY694), and quantitated pSTAT5 fluorescence intensity gated on the CD3^+^ T-cell population. For IFNγ-induced STAT1 phosphorylation, cells were stained with PE anti-human CD14 (Beckman Coulter, Indianapolis, IN, USA) and Alexa Fluor 647 anti-STAT1 (pY701), and quantitated pSTAT1 fluorescence intensity gated on CD14^+^ monocytes/macrophages.

### Mouse bone marrow macrophage-derived osteoclastogenesis

Bone marrow cells were obtained from C57BL/6 mouse tibiae and suspended in culture medium supplemented with monocyte colony-stimulating factor (100 ng/ml) for 16 hours. Nonadherent cells were harvested and further cultured with monocyte colony-stimulating factor (M-CSF) (100 ng/ml) and receptor activator of nuclear factor kappa-B ligand (100 ng/ml) for 3 days to induce the formation of multinuclear osteoclasts [10-13]. The cells were stained using a tartrate-resistant acid phosphate (TRAP) staining kit (Sigma, St Louis, MO, USA). TRAP^+^ multinuclear cells were counted for each well under a microscope.

### Toll-like receptor 9-mediated B-cell activation and plasmablast differentiation

Human B cells were enriched using RosetteSep® human B-cell enrichment cocktail (StemCell Technologies, Vancouver, Canada), followed by stimulation with ODN2006 (1 μM; InvivoGen, San Diego, CA, USA) and IFNα (20 ng/ml) for 3 days. The IL-6 production in the supernatant was measured by AlphaLISA kit (Perkin Elmer). The live cells were quantitated by the CellTiter-Glo® luminescent Cell Viability Assay kit (Promega, Madison, WI, USA).

Human B cells were differentiated with ODN2006 (50 nM) and IL-2 (10 ng/ml) for 6 days. The differentiated cells were stained with V450 anti-CD38, FITC anti-CD20, PE anti-CD19 and APC intracellular IgM. The plasmablasts were identified as CD19^+^CD38^+^CD20^–^IgM^+^ cells. The production of IgG and IgM was quantitated by AlphaLISA.

### Toll-like receptor 9-mediated plasmacytoid dendritic cell activation

Human plasmacytoid dendritic cells (pDCs) were isolated by negative selection from PBMCs with the human pDC Isolation Kit (Miltenyi Biotec, Auburn, CA, USA). The purity was confirmed with CD303 (BDCA-2; Miltenyi Biotec) staining and stimulated with ODN2216 (1 μM) for 2 days. The production of IFNα and TNFα was measured by AlphaLISA.

### Murine collagen-induced arthritis model

The mCIA model has been reported previously [9]. Briefly, DBA1/J male mice were injected intradermally with 0.1 ml bovine type II collagen (100 μg; Chondrex Inc., Redmond, WA, USA) and complete Freund’s adjuvant (200 μg; Difco, Detroit, MI, USA) followed by second immunization on day 21 with bovine type II collagen and incomplete Freund adjuvant. RO9021 was administered orally, randomized into different groups (14 mice/groups), every day for 14 days starting on the day after second immunization. Clinical arthritis scores (1 to 4) of individual paws were assessed and the arthritic index for each mouse was determined by adding the individual scores of all four paws. The level of cytokines in serum was determined by Luminex analysis (Millipore, Billerica, MA, USA).

### Histopathological analysis

Hind paws from CIA mice were collected into 10% neutral buffered formalin. After decalcification in 10% formic acid, paws were embedded in paraffin, sectioned at 8 μm and stained with toluidine blue. Inflammation (infiltration of inflammatory cells), pannus, cartilage damage, and bone resorption were scored in a double-blinded fashion by a board-certified pathologist at Boulder BioPATH, Inc. (Boulder, CO, USA) using standard criteria, with 0 being normal and 5 being the most severe.

### Half-maximal inhibitory concentration determination and statistical analysis

Half-maximal inhibitory concentration (IC_50_) values and dose/concentration response curves were determined by sigmoidal dose–response curve fitting using XLFit (IDBS, Alameda, CA, USA) or Prism (GraphPad Inc., La Jolla, CA, USA). In most studies, the IC_50_ values reported were the average from at least two studies conducted with samples in replicate. For *in vivo* studies, one-factor and two-factor comparisons were performed, respectively, using one-way or two-way analysis of variance plus Dunnett’s post test.

## Results

### Biochemical characterization of RO9021, a potent and selective SYK inhibitor

**Figure 1:**
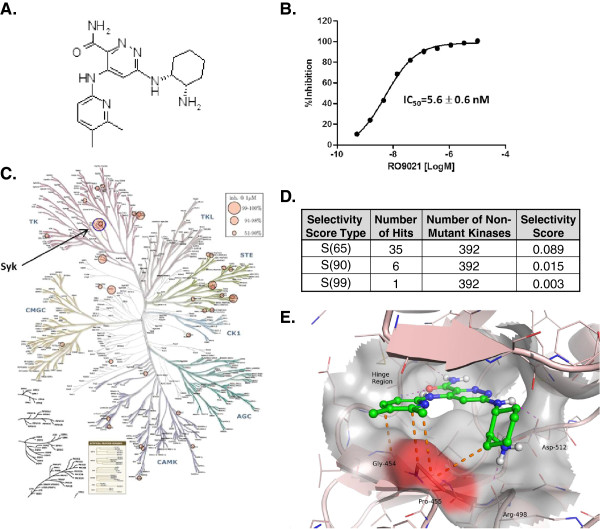
**Structure, potency and selectivity of a novel spleen tyrosine kinase inhibitor, RO9021. (A)** Compound structure of RO9021, 6-((1R,2S)-2-amino-cyclohexylamino)-4-(5,6-dimethyl-pyridin-2-ylamino)-pyridazine-3-carboxylic acid amide. **(B)** Inhibition of spleen tyrosine kinase (SYK) enzymatic activity, measured by incorporation of ^33^P-ATP into SYK substrate peptide. The half-maximal inhibitory concentration (IC_50_) is reported as the average value of three independent assays. **(C)** Kinome selectivity of RO9021. RO9021 was profiled against 392 nonmutant kinases by KinomeScan and presented as a kinome dendrogram. Circle size is proportional to percentage inhibition at the test concentration (1 μM): largest circle, 99% inhibition; medium circle, 90 to 99% inhibition; smallest circles, 51 to 90% inhibition. Arrow, SYK kinase (blue circle). **(D)** Selectivity score of RO9021. The selectivity score is a quantitative measure of compound selectivity, calculated by dividing the number of kinases that compounds bind to by the total number of distinct kinases tested, excluding mutant variants. **(E)** Structural basis of RO9021 selectivity. Crystal structure of RO9021 bound to SYK. Orange dotted lines, possible hydrophobic interactions between RO9021 and the Pro455/Gly454 region (surface shaded red).

RO9021 (Figure 1A) was identified following extensive medicinal chemistry optimization of a lead identified from high-throughput screening of Roche’s proprietary chemical compounds library. In a SYK kinase enzymatic assay, RO9021 potently inhibited SYK kinase activity with an average IC_50_ of 5.6 nM (Figure 1B). Selectivity of RO9021 against a panel of 451 wild-type and mutant protein kinases was assessed using an ATP binding site competition assay developed by KINOMEscan Inc. [3]. As shown in the dendrogram depicting a qualitative overall impression of kinase selectivity, RO9021 was highly selective for SYK enzyme (largest circle, marked blue) at 1 μM concentration (Figure 1C). The selectivity of RO9021 was quantitatively expressed as a selective score (S-score), which was calculated by dividing the number of RO9021-bound kinases by the total number of wild-type protein kinases tested (*n* = 392), excluding mutant variants. The S-score is an unbiased measure that enables quantitative comparisons between compounds. A lower S-score means higher selectivity [14]. As shown in Figure 1D, RO9021 is a highly selective SYK inhibitor with low S-scores of 0.003 for S(99) and 0.015 for S(90), indicating that SYK is the only kinase with 99% competition with RO9021 in a total of 392 tested kinases. There were only a total of seven kinases, including SYK, having more than 90% competition with RO9021 (listed in Additional file 1: Figure S1).

The expected binding mode of RO9021 was confirmed by the determination of the co-crystal structure of RO9021 and the SYK protein kinase domain (Figure 1E; Additional file 1: Figure S2). The cis-cyclohexyldiamino moiety of RO9021 formed a hydrogen bond via its secondary amine with the carboxy side chain of D512 of SYK, while the primary amine forms a hydrogen bond with the backbone of Arg498 and a salt bridge with the other oxygen of the D512 side chain. The 5,6-dimethylpyridine group of RO9021 projected out over to Gly454 and Pro455, making hydrophobic contacts. A proline at this position (Pro455) in the ATP binding site is rare in kinases, present in only nine out of a total of 433 kinases, so these interactions probably contribute to the high selectivity of this compound for SYK [15].

### RO9021 selectively suppresses B-cell receptor signaling

**Figure 2:**
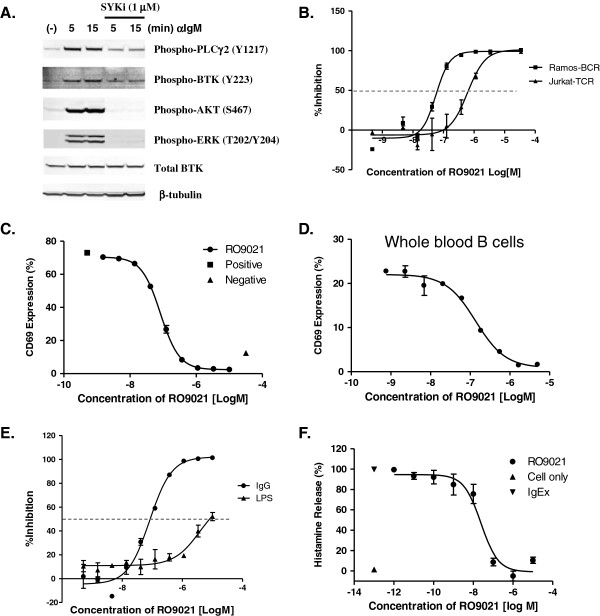
**Inhibition of B-cell receptor and Fc Receptor pathways by RO9021. (A)** RO9021 inhibited phosphorylation of PLCγ2(Y1217), BTK(Y223), AKT(S476) and ERK(p42/44) (T202/Y204) in anti-IgM stimulated Ramos cells. The levels of total Bruton’s tyrosine kinase (BTK) and β-tubulin were used as loading controls. **(B)** RO9021 suppressed anti-IgM mediated calcium flux in Ramos cells (Ramos-BCR, squares), but not anti-CD3-mediated calcium flux in Jurkat cells (Jurkat-TCR, triangles). Intracellular calcium concentrations were measured using a calcium-sensitive fluorescent dye and presented as calculated percentage inhibition. **(C)**, **(D)** Inhibition of anti-IgM-induced CD69 expression in human peripheral blood mononuclear cells (PBMCs) **(C)** and whole blood **(D)**. Data were in duplicate and are shown as mean ± standard deviation (SD). **(E)** RO9021 inhibited FcγR signaling (human IgG-coated beads, dots), but not toll-like receptor-4 (TLR4)-induced TNFα production (lipopolysaccharide (LPS), triangles) in human monocytes. Percentage of inhibition by compound presented. Data were in quadruplicate and are shown as mean ± SD. **(F)** Inhibition of histamine release in human mast cells induced by anti-4-hydroxy-3-nitrophenylacetyl hapten (NP) IgE and NP. Anti-NP IgE and NP cross-linking-induced a robust histamine release (cells alone, triangle; IgEx, inverted triangle). RO9021 attenuated IgE-NP-induced histamine release in a concentration-dependent manner (circle). Data were in triplicate and are shown as mean ± SD.

Since SYK is best studied as a key mediator of BCR activating signals within B cells, we first evaluated the effect of RO9021 in blocking BCR-dependent responses. The human B-cell line, Ramos, was pretreated with 1 μM RO9021 prior to anti-IgM antibody-induced cross-linking of the BCR. The activation of various BCR signaling components was assessed by western blot using phospho-specific antibodies. As shown in Figure 2A, treatment with RO9021 inhibited anti-IgM induced phosphorylation of BTK, PLCγ2, AKT and ERK, indicating that blockade of SYK kinase activity by RO9021 resulted in attenuation of BCR downstream signaling cascade.

Next, we examined the effect of RO9021 in several functional outcomes of BCR signaling using both human B-cell lines and primary cells. Consistent with the known biology of SYK, RO9021 blocked anti-IgM-activated calcium flux in Ramos B-cell line with an IC_50_ value of 78 ± 21 nM. This effect is specific to BCR signaling because RO9021 showed about 12-fold potency shift (IC_50_ = 910 ± 270 nM) in blocking T-cell receptor (anti-CD3)-induced calcium flux in Jurkat, a human T-cell line (Figure 2B). Finally, when tested in human PBMCs or whole blood, RO9021 inhibited BCR-dependent cell surface CD69 expression in CD20^+^ B cells with IC_50_ values of 83 nM and 87 nM, respectively (Figure 2C,D).

### Selective inhibition of Fc receptor signaling in monocytes and mast cells by RO9021

SYK is also recruited into activated Fc receptor through an interaction with the phosphorylated ITAM motifs of the receptor and mediates Fc receptor downstream signaling. We therefore examined the effects of inhibiting SYK kinase activity with RO9021 on FcγR signaling in human monocytes and FcϵR signaling in human mast cells. As shown in Figure 2E, the production of the proinflammatory cytokine TNFα induced by crosslinking of FcγR on human monocytes was inhibited by RO9021 with an IC_50_ value of 63 ± 19 nM. In contrast, RO9021 had very weak effect on Toll-like receptor (TLR)-4-dependent TNFα production (IC_50_ = 2.9 ± 0.9 μM) in monocytes stimulated by lipopolysaccharide, indicating that RO9021 blocks the FcγR pathway in a specific manner. Furthermore, RO9021 also displayed a similar inhibitory potency (IC_50_ = 22.8 ± 1.7 nM) in a FcϵR-mediated mast cell activation and degranulation assay, as judged by inhibition of IgE/antigen-induced histamine release (Figure 2F).

### RO9021 does not appreciably inhibit the JAK–STAT pathway

**Figure 3:**
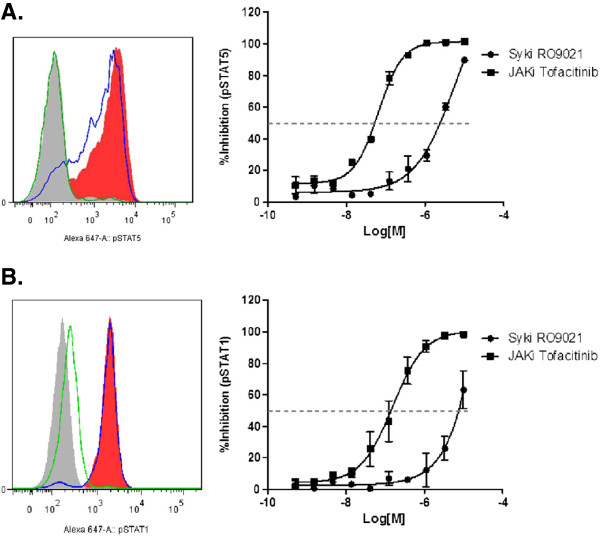
**Marginal effect of RO9021 in the JAK–STAT pathway.** Human peripheral blood mononuclear cells were pretreated with RO9021 or the janus kinase (JAK) inhibitor tofacitinib and then stimulated with IL-2 to induce STAT5 phosphorylation in CD3^+^ T cells **(A)** or IFNγ to induce STAT1 phosphorylation in CD14^+^ monocytes/macrophages **(B)**. Left panels, representative overlaid histograms of flow cytometric analysis. Filled gray, unstimulated; filled red, stimulated with IL-2 or IFNγ. Blue line, treatment with 1 μM RO9021; green line, treatment with 1 μM JAK inhibitor (tofacitinib). Right panels: concentration-dependent inhibition curves of RO9021 (dots) and tofacitinib (squares).

In addition to SYK, the KINOMEscan analysis revealed that there were six other kinases with more than 90% competition with RO9021 (Additional file 1: Figure S1). Of particular interest are JAK1 and JAK3 because pharmaceutical inhibitors of these family members have demonstrated clinical efficacy in RA trials. We therefore examined whether RO9021 had any cellular JAK activity. To that end, PBMCs were pretreated with RO9021 prior to stimulation with either IL-2 to activate the JAK1/3–STAT5 pathway in T cells or IFNγ to activate the JAK1/2–STAT1 pathway. As a positive control, the JAK inhibitor tofacitinib (CP 690550) was included in the analysis. As shown in Figure 3A, phospho-STAT5 staining in the CD3^+^ T-cell population was induced by IL-2 stimulation (filled red histogram vs. nonstimulated filled gray histogram). At 1 μM, tofacitinib completely blocked the phosphorylation of STAT5 (green line) whereas RO9021 had no significant effect (blue line). Concentration-dependent inhibitory curves (Figure 3A, right) also showed significant potency shift between tofacitinib and RO9021, with average IC_50_ values of 31 ± 13 nM and 1.32 ± 0.66 μM, respectively. Similarly, in IFNγ-treated CD14^+^ monocytes, RO9021 had no effect on the phosphorylation of STAT1, except at the highest concentration tested (10 μM) (Figure 3B). In contrast, tofacitinib inhibited STAT1 phosphorylation in a concentration-dependent manner with an average IC_50_ value of 81 ± 50 nM. RO9021 thus did not appear to appreciably inhibit the JAK–STAT pathway in the cell, further supporting the selectivity of the compound.

### SYK kinase activity is essential for osteoclastogenesis

**Figure 4:**
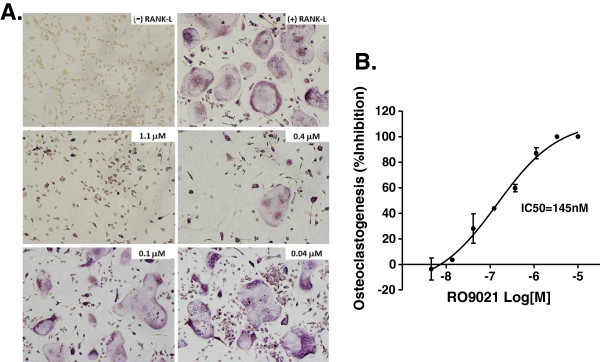
**Dose-dependent inhibitory effects of RO9021 on osteoclastogenesis. (A)** Representative images of tartrate-resistant acid phosphate (TRAP) staining showing receptor activator of nuclear factor kappa-B ligand (RANK-L) and monocyte colony-stimulating factor (M-CSF) mediated osteoclastogenesis from mouse bone marrow macrophages (BMM). Osteoclasts were shown as TRAP^+^ multinuclear cells. Negative and positive controls labeled as (–) RANK-L (upper left) and (+) RANK-L (upper right), respectively. The concentration of RO9021 was labeled on the upper right corner of each image. **(B)** TRAP^+^ multinuclear osteoclasts were counted under a microscope and percentage of inhibition on osteoclastogenesis was presented. Results were in triplicate and are plotted as mean ± standard deviation. IC_50_, half-maximal inhibitory concentration.

Bone destruction is one of the hallmarks of RA and is mainly attributed to an abnormal activation and differentiation of macrophages into osteoclasts that mediate bone erosion [1,16-18]. We therefore assessed the effects of RO9021 on osteoclastogenesis using mouse bone marrow-derived macrophages. Mouse bone marrow macrophages were differentiated by treatment with soluble receptor activator of nuclear factor kappa-B ligand and monocyte colony-stimulating factor in the presence of RO9021 for about 3 days. As shown in Figure 4, exposure to RO9021 abrogated the formation of multinuclear TRAP^+^ osteoclasts in a concentration-dependent manner; TRAP^+^ cells were barely detectable in the presence of more than 0.4 μM RO9021. The results suggest inhibition of SYK kinase activity may prevent bone erosion in arthritis, which is consistent with previous *SYK* knockout mice studies [19-23].

### RO9021 reveals a novel role for SYK in Toll-like receptor 9-dependent signaling

As BTK has been implicated in TLR signaling [24,25], we next sought to explore the kinase function of SYK in TLR9-mediated responses in human B cells and pDCs with selective SYK inhibitor.

**Figure 5:**
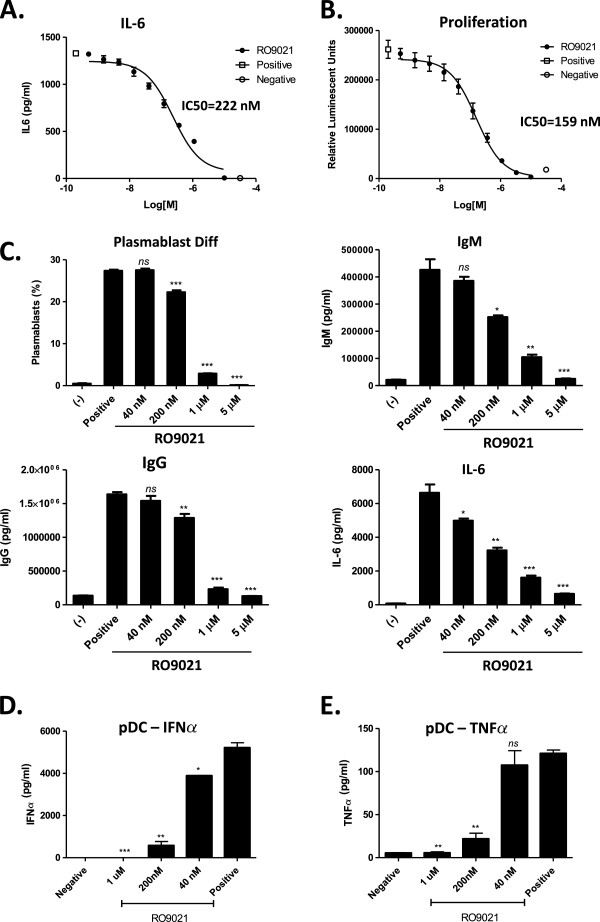
**Inhibition of Toll-like receptor-9 pathway in human B cells and plasmacytoid dendritic cells by RO9021. (A), (B)** Human B cells were stimulated with CpG-B (ODN2006) and IFNα for 2 days. Production of IL-6 **(A)** and B-cell proliferation **(B)** was blocked by RO9021. The proliferation was presented by total alive cells, which were quantitated by Celltiter Glow as relative luminescent units. **(C)** RO9021 inhibited human plasmablast differentiation. Human B cells were differentiated with ODN2006 and IL-2 for 6 days. The plasmablasts were identified as CD19^+^CD38^+^CD20^–^ cells and presented as the percentage in total CD19^+^ B cells. The levels of IgM, IgG and IL-6 in the supernatants were also blocked by RO9021. **(D)**, **(E)** RO9021 blocked Toll-like receptor 9 (TLR9)-mediated cytokine production in human plasmacytoid dendritic cells (pDCs). Purified pDC cells were stimulated with CpG-A (ODN2216) for 2 days. The production of IFNα **(D)** and TNFα **(E)** in supernatant were determined. Data were in duplicate and are shown as mean ± standard deviation. Statistical analysis of each treatment compared with the positive group was performed by Student’s *t* test. *P <0.05; **P <0.01; ***P <0.001; ns, not statistically significant. Plots shown are representative of four independent experiments tested in different donors. IC_50_, half-maximal inhibitory concentration.

Interestingly, we found that the kinase function of SYK was important for mediating TLR9 responses. As demonstrated previously, IFNα when used in combination with TLR9 ligand, ODN2006, synergistically activated B cells as measured by the production of IL-6 [26,27], which was inhibited by RO9021 (Figure 5A; IC_50_ = 222 ± 47 nM). In addition, TLR9-dependent B-cell proliferation was also dose-dependently inhibited by RO9021 (Figure 5B; IC_50_ = 159 ± 28 nM).

We next differentiated B cells with CpG (ODN2006) and IL-2 in the presence of different concentrations of RO9021. After 6 days of culture, a significant percentage of B cells (~30%) proliferated and differentiated into CD19^+^CD38^+^CD20^–^ and intracellular IgM-positive plasmablast cells. Treatment with RO9021 blocked the generation of plasmablast cells in a concentration-dependent manner (Figure 5C). Consistent with the observed decreased percentage of plasmablast cells, the production of IgM, IgG, and IL-6 in the supernatant was also reduced by RO9021 (Figure 5C).

As pDCs are the main source for IFNα, we next examined the effect of RO9021 on TLR9-mediated IFNα production in pDCs. Purified human pDCs (CD303^+^) were stimulated with ODN2216 for 2 days and the levels of IFNα and TNFα were measured. As shown in Figure 5D,E, IFNα was highly produced by pDCs upon TLR9 activation, relative to the small amount of TNFα detected. Importantly, RO9021 inhibited the production of both cytokines in a concentration-dependent fashion.

### RO9021 inhibits progression of murine collagen-induced arthritis

Based on the above findings that SYK inhibition by RO9021 is able to impinge on several innate and adaptive immune responses, we speculated that the compound should have therapeutic efficacy in an autoimmune disease model. Furthermore, RO9021 showed reasonable *in vivo* pharmacokinetic profiles after single oral administration (Table 1 and Additional file 1: Figure S3). No significant inhibitions of CYP450 isozymes (IC_50_ >50 μM) and hERG (IC_20_ = 5.5 μM) were observed at pharmacological concentrations (data not shown).

**Table 1:** Pharmacokinetics profile of single oral dosing RO9021 in mouse

**Dose**	**C**_**max **_**(nM)**	**T**_**max **_**(hours)**	**T**_**1/2 **_**(hours)**	AUC (hour*nM)
45 mg/kg	5,763.69	0.50	3.54	35,804.26
5 mg/kg	694.52	1.00	4.34	3,847.84

C_max_, maximum plasma concentration; T_max_, time after administration of a drug when the maximum plasma concentration is reached; T_1/2_, half-life; AUC, area under the curve.

**Figure 6:**
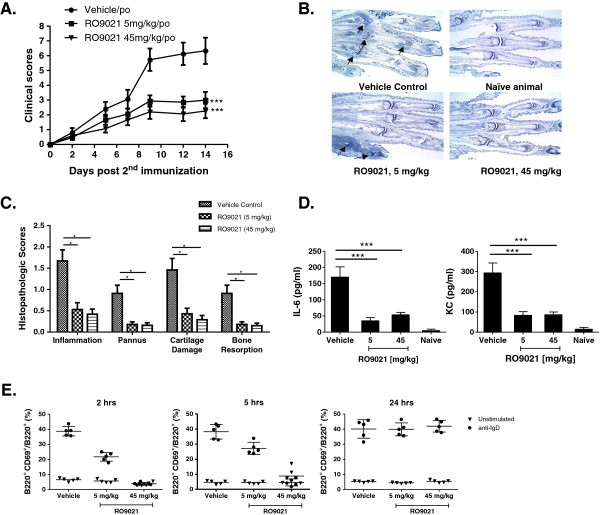
**Oral administration of RO9021 abrogated collagen-induced arthritis in mice. (A)** Dose-dependent attenuation of clinical scores by RO9021 (*n* = 14/group). ***P <0.001 compared with vehicle-treated group. po, per orally. **(B)** Representative toluidine blue-stained paraffin section of hind paws of vehicle (left upper), 5 mg/kg RO9021 (left bottom) and 45 mg/kg RO9021 (right bottom) treated mice. Photomicrograph of naïve mice also shown (right upper). Intense blue staining within joint space (arrows) indicated representative affected joints with inflammation and pannus formation. **(C)** Histopathological quantitation of inflammation, pannus formation, cartilage damage, and bone resorption in the hind paws of a subgroup of mice (*n* = 15/group). *P <0.05*.***(D)** Serum level of IL-6 and KC(CXCL1) after 14 days of treatment with vehicle or RO9021. ***P <0.001 compared with vehicle-treated group. **(E)** Inhibition of anti-IgD induced CD69 expression in *ex vivo* whole blood assay at 2 and 5 hours post dose, but not at 24 hours post dose. CD69 expression in B220^+^ cells was *induced ex vivo* with anti-IgD in terminal whole blood from subgroups of mice. CD69 expression in unstimulated blood (inverted triangle) was also examined and used as baseline control (*n* = 5/group).

To this end, we evaluated RO9021 in the mCIA model of RA. As shown in Figure 6A, RO9021 administered orally at 5 and 45 mg/kg daily, starting on the day of the second immunization (that is, day 21), for 14 days inhibited arthritis progression in a dose-dependent manner as measured by the clinical scores. There was significant efficacy on arthritis in both the 5 and 45 mg/kg dosing groups compared with the vehicle group. As shown in photomicrographs (Figure 6B) and quantitation (Figure 6C) from histopathological analysis, vehicle-treated, but not RO9021-treated, mice had severe inflammation and cartilage damage with pannus and resorption in the ankle and all digit joints. Notably, measured levels of cytokines IL-6 and KC(CXCL1) in mouse serum were also markedly reduced after 14 days of treatment with RO9021 (Figure 6D).

To demonstrate on-target inhibition by RO9021 and the pharmacokinetics and pharmacodynamics relationship, mouse blood samples were collected at 2, 5 and 24 hours post compound dosing. As shown in Figure 6E, pharmacodynamics effects based on cell-surface CD69 expression on B cells (B220^+^), as judged by *ex vivo* stimulation with anti-IgD, were consistent with pharmacokinetics analysis of compound exposure. RO9021 inhibited anti-IgD-induced CD69 expression on B cells at 2-hour and 5-hour time points, but not at the 24-hour time point, suggesting 5-hour compound coverage was sufficient to significantly impact disease progression in this model.

## Discussion

Current biologic agents have not been able to break the ceiling in terms of delivering better and broader efficacy for treatment of autoimmune diseases. Given this persistent unmet need and the promise of multipathway inhibition to deliver breakthrough efficacy, pharmacological modulation of intracellular signaling components with small molecule agents offers an attractive alternative therapeutic strategy, provided the risk/benefit profile is acceptable. In this regard, the SYK–BTK axis is an attractive target because it is critical for antigen receptor signaling, abnormal regulation of which has been implicated in the pathogenesis of several autoimmune diseases, including RA and SLE [28].

Among the reported agents targeting the SYK, the inhibitor fostamatinib (R788) has demonstrated reduced clinical efficacy compared with other therapeutic agents. In our hands, however, R788 is not a very selective kinase inhibitor, inhibiting one-half of the kinome in the KinaseScan assay, including JAK and vascular endothelial growth factor receptor (data not shown), which is consistent with previous reports [15,29], suggesting that the clinical activities of R788 are not solely attributed to SYK inhibition. Some of the off-target activities might also account for the observed adverse effects in clinical trials, including high blood pressure, which is due to vascular endothelial growth factor receptor inhibition. R788 is also a relatively weak SYK inhibitor in whole blood assays, which is potentially attributed to high plasma protein binding. A more selective, potent SYK inhibitor will thus be necessary to address the mechanism of action and evaluate the efficacy as well as any potential on-mechanism toxicity associated with SYK inhibition in clinical trials.

To this end, we have developed an alternative chemical scaffold of SYK inhibitor, designated RO9021. The protein kinase selectivity profile of RO9021 was assessed by the widely accepted KinomeScan method, which utilizes a proprietary active site-directed competition binding assay to quantitatively measure interactions between test compounds and more than 450 human kinases and disease-relevant mutant variants. As shown in Figure 1 and Additional file 1: Figure S1, beside SYK with 99% competition only six protein kinases, including JAK1 and JAK3, have more than 90% competition, indicating that RO9021 has superb selectivity. Since truncated forms of recombinant JAK1 and JAK3 were utilized in the KinomeScan assay, we examined the ability of RO9021 to inhibit JAK-mediated signaling in cell-based assays and found the compound had weak or no activity (Figure 3). In contrast, RO9021 inhibited phosphorylation of SYK downstream effectors, namely PLCγ2 and BTK, in response to BCR engagement (Figure 2A), consistent with the known biology of SYK in BCR signaling. Taken together, these data strongly indicate that the compound effect in cells is mediated by SYK inhibition. Furthermore, RO9021 has reasonable oral bioavailability profiles and thus can be used to interrogate the various reported biological roles of SYK in preclinical disease models. In addition to suppression of BCR signaling in human PBMCs and whole blood (Figure 2C,D), FcγR signaling in human monocytes (Figure 2E), and FcϵR signaling in human mast cells (Figure 2F), we showed that RO9021 also blocked osteoclastogenesis of mouse bone marrow macrophages *in vitro* (Figure 4).

Certain autoimmune diseases, such RA and SLE, arise from an inappropriate immune response of the body against self-antigens [1,30,31]. SLE, for instance, is characterized by the loss of tolerance to self-nuclear antigens, the deposition of immune complexes in tissues, and multiorgan involvement [32]. Studies have shown that nuclear-acid sensing pathways implicated in the subversion of the innate immune response to discriminate between self-antigen and foreign antigens are those mediated by the TLRs in the context of SLE pathogenesis [32-34]. BTK, which is a downstream kinase of SYK, has been implicated in TLR signaling recently [24,25], whereas the role of SYK in TLR signaling is not well appreciated. It has been reported that the TLR9 agonist CpG could induce TLR-9 independent SYK phosphorylation and activation through actin cytoskeleton reorganization, leading to activation of Src family kinases [35]. Recruitment of SYK to TLR9 and phosphorylation of TLR9 are required for CpG-induced cytokine production. We therefore used RO9021 to study the role of SYK in TLR9 signaling. Interestingly, the kinase function of SYK is essential for TLR9-mediated responses in human B cells (Figure 5A,B,C). Inhibition of SYK kinase function resulted in a decreased level of plasmablasts, IgM, IgG and IL-6 upon B-cell differentiation in the presence of TLR9 ligand. In addition, RO9021 also potently inhibited IFNα production by human pDCs upon TLR9 activation (Figure 5D). Importantly, the effects on TLR9 responses are specific because RO9021 did not inhibit TLR4-dependent TNFα production by human monocytes (Figure 2E) or activation of the JAK–STAT pathway stimulated with either IL-2 or IFNγ (Figure 3). Our study showed for the first time that kinase activity of SYK is critical for TLR9 signaling pathway in B cells and pDCs.

The role of SYK in TLR signaling in B cells and pDCs could have significant implication for SYK inhibitors as therapeutic agents for SLE since the development and progression of the disease are believed to be driven by the inappropriate activation of TLR7, TLR8 and TLR9. Other studies have indicated that autoimmunity in RA and psoriasis also is mediated through one or more of these TLRs [36-41]. In this regard, it is noteworthy that TLR antagonists such as IRS-954 (TLR7 and TLR9 antagonist) [42,43] and IMO-8400 (TLR7, TLR8 and TLR9 antagonist) are currently undergoing clinical trials in SLE.

Consistent with its inhibitory activities in various innate and adaptive immune responses, oral administration of RO9021 inhibited arthritis progression in the mCIA model (Figure 6). Importantly, there was correlation between pharmacokinetics analysis of compound exposure and pharmacodynamics analysis based on anti-IgD-induced CD69 expression on B cells (B220^+^), indicating on-target mode of action. Furthermore, the pharmacodynamics analysis suggests that 5-hour compound coverage was sufficient to ameliorate the disease in this model.

## Conclusions

The present study collectively suggests RO9021 is a selective, potent and orally bioavailable small molecule SYK kinase inhibitor, which could serve as a promising chemical lead for the design of clinical SYK inhibitors and could complement the current arsenal of tools in development for treatment of inflammation-related and autoimmune-related disorders.

## Abbreviations

APRIL: A proliferation-inducing ligand; BAFF: B-cell activating factor; BCR: B-cell receptor; BTK: Bruton’s tyrosine kinase; CIA: Collagen-induced arthritis; IC50: Half-maximal inhibitory concentration; IL: Interleukin; JAK: Janus kinase; M-CSF: Monocyte colony-stimulating factor; PBMC: Peripheral blood mononuclear cell; pDC: Plasmacytoid dendritic cell; RA: Rheumatoid arthritis; SLE: Systemic lupus erythematosus; S-score: Sensitivity score; SYK: Spleen tyrosine kinase; TLR: Toll-like receptor; TNF: Tumor necrosis factor; TRAP: Tartrate-resistant acid phosphate.

## Competing interests

All authors were employees of Hoffmann-La Roche during the preparation of this manuscript, and declare that they have no competing interests.

## Authors’ contributions

CL designed and coordinated the study, carried out the experiments and interpretation of the results, and wrote the manuscript. JH, JZ, AP, JM, TW, NR, TT, MS, CL, YK, D-QH, and DX carried out or participated in the experiments. MZ worked on pharmacokinetics data. JH and ML designed the compound. SN and JD supervised the study. S-LT supervised the study and wrote the manuscript. All authors read and approved the final manuscript.

## Supplementary Material

Additional file 1 **Figure S1 lists kinases with more than 90% binding efficiency with RO9021 (1 μM) in the KinomeScan assay.** Figure S2 shows the X-ray crystal structure of SYK with front view (A) and top view (B), showing the ATP binding site with RO9021 bound. Black dashes, hydrogen bonds; orange dashes, hydrophobic contacts to the Gly454/Pro455 region. Figure S3 shows the pharmacokinetics profile of single oral dose of RO9021 in mouse. Click here for file
