# Supplementary material for: Selective inhibition of spleen tyrosine kinase (SYK) with a novel orally bioavailable small molecule inhibitor, RO9021, impinges on various innate and adaptive immune responses: implications for SYK inhibitors in autoimmune disease therapy
Source: Arthritis Res Ther. 2013 Oct 4;15(5):R146. doi: 10.1186/ar4329 (PMC3978604; doi:10.1186/ar4329)
Supplement: Additional file 1 — Figure S1 lists kinases with more than 90% binding efficiency with RO9021 (1 μM) in the KinomeScan assay. Figure S2 shows the X-ray crystal structure of SYK with front view (A) and top view (B), showing the ATP binding site with RO9021 bound. Black dashes, hydrogen bonds; orange dashes, hydrophobic contacts to the Gly454/Pro455 region. Figure S3 shows the pharmacokinetics profile of single oral dose of RO9021 in mouse. [file ar4329-S1.pdf]

Supplementary Figure S1.

Kinase Target	%Competition
SYK	99
JAK1(JH2domain-pseudokinase)	97
JAK3(JH1domain-catalytic)	97
GCN2(Kin.Dom.2,S808G)	95
SLK	93
FLT3(ITD)	92
PAK7	92

Figure S1. Listed kinases with more than 90% binding efficiency with RO9021 (1 μM) in KinomeScan Assay.

Supplementary Figure S2.

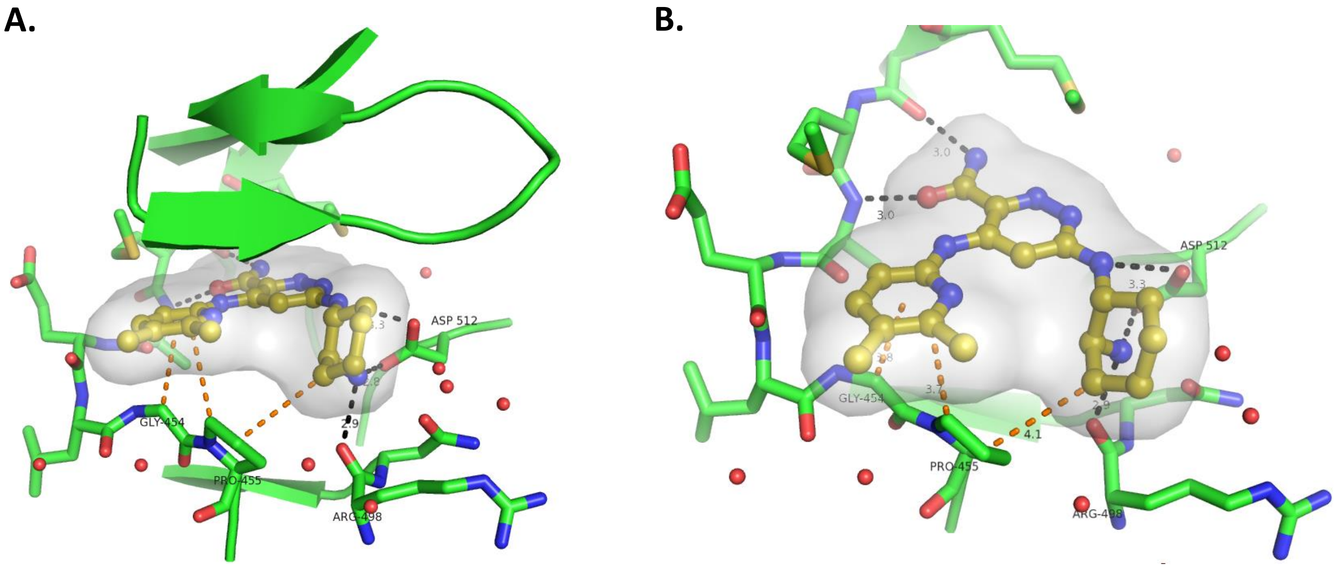

Supplemental Fig. S2. The X-ray crystal structure of SYK with front view (A.) and top view (B.), showing the ATP binding site with RO9021 bound. Black dashes represent hydrogen bonds, and orange dashes show hydrophobic contacts to the Gly454/Pro455 region.

Supplementary Figure S3.

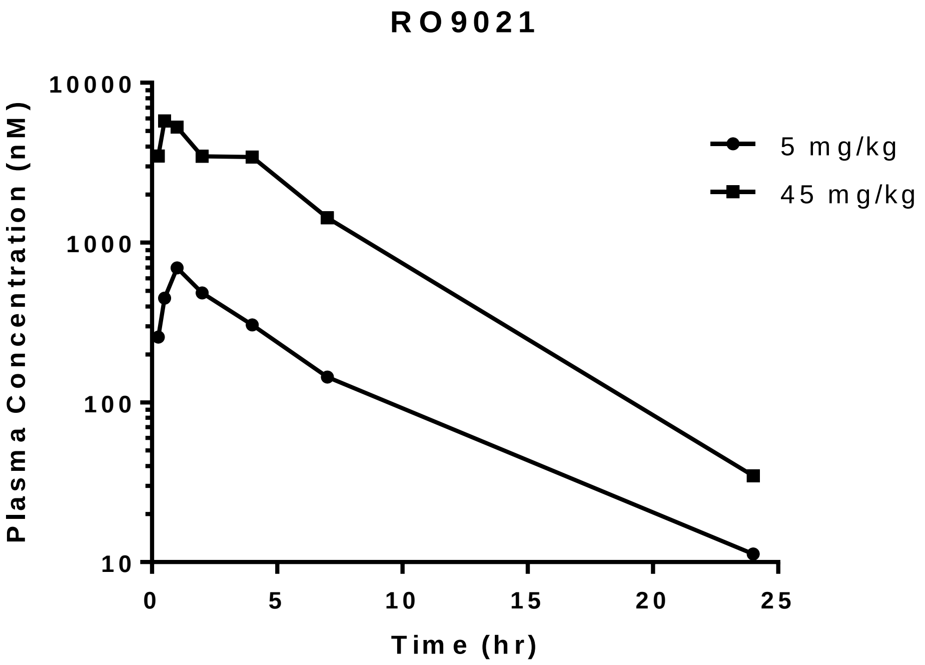

Supplemental Fig. S3. Pharmacokinetics (PK) profile of single oral dose of RO9021 in mouse.
